# Supplementary material for: Noc1 downregulation induces nucleolar stress and upregulates p53 isoforms, with a robust increase of the truncated p53E isoform in Drosophila wing discs
Source: G3 (Bethesda). 2026 Jan 14;16(3):jkaf313. doi: 10.1093/g3journal/jkaf313 (PMC12958821; doi:10.1093/g3journal/jkaf313)
Supplement: jkaf313_Supplementary_Data [file jkaf313_supplementary_data.zip › Supplementary_Figure_2_G3-2025-406304.pdf]

Supplementary Figure 2

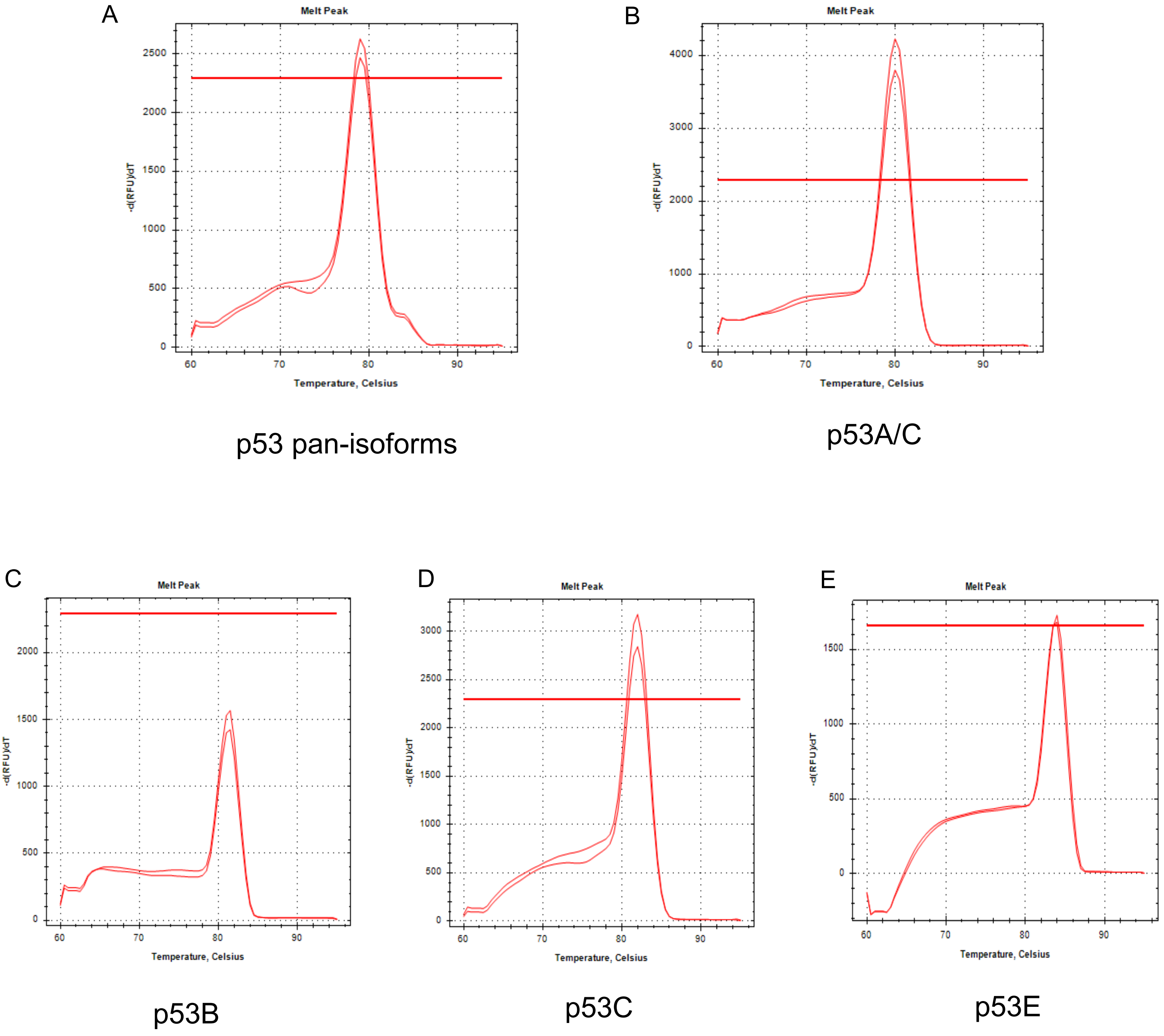

Supplementary Figure 2. Melting curves of the p53 primers isoforms described in this work.

(A-E) Melting curves of the indicated primers obtained after qPCR on mRNA extracted from wing imaginal discs from third instar larvae from *rotund-Gal4 with UAS-Lac-z-RNAi* crosses in the w^{1118} background. For every pair of primers, only one peak is present. qPCR was done with Bio-Rad CFX96, and melting curves were obtained using Bio-Rad CFX Manager software.
