# Supplementary material for: Noc1 downregulation induces nucleolar stress and upregulates p53 isoforms, with a robust increase of the truncated p53E isoform in Drosophila wing discs
Source: G3 (Bethesda). 2026 Jan 14;16(3):jkaf313. doi: 10.1093/g3journal/jkaf313 (PMC12958821; doi:10.1093/g3journal/jkaf313)
Supplement: jkaf313_Supplementary_Data [file jkaf313_supplementary_data.zip › Supplementary_Figure_Legends_G3-2025-406304.docx]

**Supplementary Figure Legends**

**Supplementary Figure 1.** Sequences of all p53 primers and their relative binding sites on each isoform.

**Supplementary Figure 2.** Melting curves of the p53 isoform-specific primers designed for this paper.

**Supplementary Figure 3.** Mutations in the p53A2.3 and p53B41.5 mutant lines used in this study.
